# Bacterial Community Composition and Potential Driving Factors in Different Reef Habitats of the Spermonde Archipelago, Indonesia

**DOI:** 10.3389/fmicb.2017.00662

**Published:** 2017-04-20

**Authors:** Hauke F. Kegler, Muhammad Lukman, Mirta Teichberg, Jeremiah Plass-Johnson, Christiane Hassenrück, Christian Wild, Astrid Gärdes

**Affiliations:** ^1^Tropical Marine Microbiology, Department of Biogeochemistry and Geology, Leibniz Center for Tropical Marine ResearchBremen, Germany; ^2^Faculty of Biology and Chemistry (FB 2), University of BremenBremen, Germany; ^3^Marine Science Department, Faculty of Marine Science and Fisheries, Hasanuddin UniversitySouth Sulawesi, Indonesia; ^4^Algae and Seagrass Ecology, Department of Ecology, Leibniz Center for Tropical Marine ResearchBremen, Germany; ^5^Centre for Ocean Life, National Institute of Aquatic Resources (DTU-Aqua), Technical University of DenmarkCharlottenlund, Denmark; ^6^HGF MPG Joint Research Group for Deep-Sea Ecology and Technology, Max Planck Institute for Marine MicrobiologyBremen, Germany

**Keywords:** eutrophication, 454 pyrosequencing, microbial communities, Spermonde Archipelago, bacterial pathogens

## Abstract

Coastal eutrophication is a key driver of shifts in bacterial communities on coral reefs. With fringing and patch reefs at varying distances from the coast the Spermonde Archipelago in southern Sulawesi, Indonesia offers ideal conditions to study the effects of coastal eutrophication along a spatially defined gradient. The present study investigated bacterial community composition of three coral reef habitats: the water column, sediments, and mucus of the hard coral genus *Fungia*, along that cross-shelf environmental and water quality gradient. The main research questions were: (1) How do water quality and bacterial community composition change along a coastal shelf gradient? (2) Which water quality parameters influence bacterial community composition? (3) Is there a difference in bacterial community composition among the investigated habitats? For this purpose, a range of key water parameters were measured at eight stations in distances from 2 to 55 km from urban Makassar. This was supplemented by sampling of bacterial communities of important microbial habitats using 454 pyrosequencing. Findings revealed that the population center Makassar had a strong effect on the concentrations of Chlorophyll *a*, suspended particulate matter (SPM), and transparent exopolymer particles (TEP), which were all significantly elevated at the inshore compared the other seven sites. Shifts in the bacterial communities were specific to each sampled habitat. Two OTUs, belonging to the genera *Escherichia/Shigella* (Gammaproteobacteria) and *Ralstonia* (Betaproteobacteria), respectively, both dominated the bacterial community composition of the both size fractions of the water column and coral mucus. The sampled reef sediments were more diverse, and no single OTUs was dominant. There was no gradual shift in bacterial classes or OTUs within the sampled habitats. In addition, we observed very distinct communities between the investigated habitats. Our data show strong changes in the bacterial community composition at the inshore site for water column and sediment samples. Alarmingly, there was generally a high prevalence of potentially pathogenic bacteria across the entire gradient.

## Introduction

Coastal coral reef systems in close vicinity to highly populated urban areas are often impacted by land-based activities. The Spermonde Archipelago, including its ~150 small islands, is located on a narrow, 60 km wide carbonate shelf platform in southern Sulawesi, Indonesia. The coral reefs fringing the islands are essential to sustain the livelihoods of thousands of fishermen in the archipelago as a source of income and building material for local construction (Pet-Soede et al., 2001). The Archipelago is characterized by an eutrophication gradient from nutrient-rich coastal waters to oligotrophic offshore waters (Edinger et al., 1998). Untreated sewage and pollutants from Makassar enter the system directly or via the river Jene Berang, which additionally discharges sediments and inorganic nutrients from the hinterland (Renema and Troelstra, 2001). This leads to eutrophication, one of the primary local threats to coastal marine ecosystems (Burke et al., 2011; Paerl et al., 2014). The first response to eutrophication is often an increase in phytoplankton biomass (Fabricius, 2011). The result is an increased availability of organic matter such as dissolved organic carbon (DOC) and subsequently transparent exopolymer particles (TEP; Passow, 2000; Verdugo et al., 2004; Verdugo and Santschi, 2010). High concentrations of TEP in the water column will in turn intensify aggregation and sedimentation processes due to their high stickiness (Passow, 2002; Azam and Malfatti, 2007). The sinking particles and TEP itself are rich sources of organic matter for both free-living and particle-attached bacteria in the water column (Passow and Alldredge, 1994; Kiørboe and Tang, 2003; Kramer et al., 2013).

Several studies found significant shifts in the bacterial community composition in eutrophic and organic matter rich conditions of reef waters (Meyer-Reil and Köster, 2000; Weinbauer et al., 2010; de Voogd et al., 2015), microbial biofilms (Sawall et al., 2012; Witt et al., 2012), and sediments (Uthicke and McGuire, 2007) often alongside an increase in total bacterial cell counts (Zhang et al., 2007, 2009; Dinsdale et al., 2008). The changes are often related to a transition from autotrophic to heterotrophic bacterial communities (Meyer-Reil and Köster, 2000; Witt et al., 2012). In the water column there are two groups of bacteria, “free-living” and “particle-attached,” which use different carbon sources and are both influenced differently by changes in water quality (Becquevort et al., 1998; Zhang et al., 2007). Understanding the response of microbial communities in different coral reef habitats to spatial gradients in eutrophication is of great importance in the context of increasing anthropogenic perturbations to coastal water quality in the Spermonde Archipelago. As they play such an important role in biogeochemical cycling and coral reef health, small shifts in microbial communities, induced by increased anthropogenic eutrophication, can further alter nutrient cycling, sedimentation, and organic matter export as well as promoting coral pathogens (Bruno et al., 2003; Fabricius, 2005; Lyons et al., 2010).

To date, there are only two studies of the Spermonde Archipelago that have focused on bacterial diversity of settlement tile biofilms (Sawall et al., 2012) and bacterial communities from different reef habitats, specifically within sponges and the functional role of the associated bacteria (Cleary et al., 2015). Our study now further examines the relationship between bacterial communities, habitats, and water quality gradients in the Spermonde Archipelago, and additionally includes mucus of the common hard corals genus *Fungia* as an important bacterial habitat (Wild et al., 2004; Allers et al., 2008). We also included TEP, to the best of our knowledge, as the first study in the Spermonde Archipelago. TEP is an important, but frequently overlooked, biogeochemical water quality parameter that is pivotal for the organic matter composition and transition from the dissolved to the particulate fraction (Passow, 2002). Its formation is tightly linked to the interaction of phytoplankton with bacteria (Gärdes et al., 2011). With those properties, TEP may significantly alter bacterial communities and the ecosystem functions they provide (Passow, 2002; Buchan et al., 2014; Taylor et al., 2014). Through this multifaceted approach, including the eutrophication-related parameters and bacterial communities from different habitats, we wanted to elucidate if the relative abundance of different bacterial phylogenetic groups shift in response to changes in environmental and water quality parameters including: pH, salinity, inorganic nutrient availability, Chl a, DOC, TEP, and SPM along the eutrophication gradient. The main research questions were: (1) How do water quality and bacterial community composition change along a coastal shelf gradient? (2) Which water quality parameters influence bacterial community composition? (3) Is there a difference in bacterial community composition among the investigated habitats?

## Materials and methods

### Study sites

**Figure 1:**
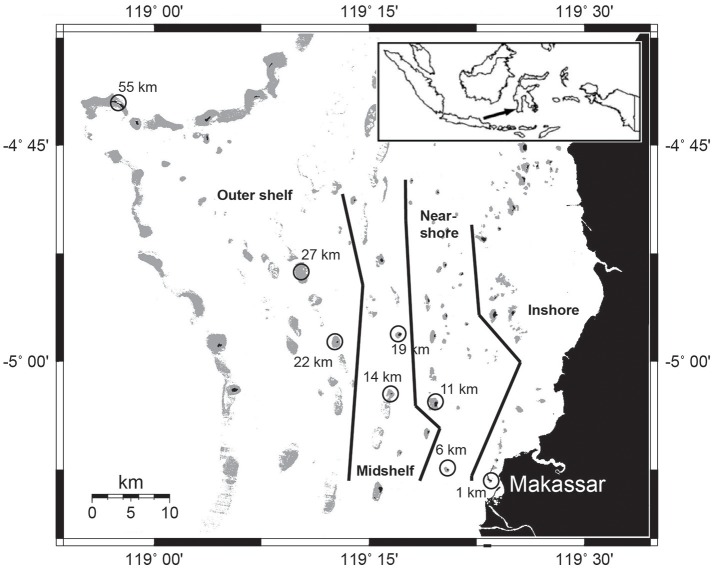
**Map of the Spermonde Archipelago in southern Sulawesi**. Sampling stations are circled with the according distance to Makassar. Map modified from Glaser et al. (2010), zonation modified from Renema and Troelstra (2001).

Sampling was carried out at eight islands across the continental shelf of the Spermonde Archipelago in South Sulawesi, Indonesia after the wet monsoon season in February 2013 (Figure 1). Due to environmental and ecological variability across the shelf, the archipelago has been divided into several ecological zones running parallel to the coast line (Moll, 1983; Renema and Troelstra, 2001). The chosen sites represent varying exposure to eutrophication from an inshore station in close proximity (1 km) to metropolitan Makassar (population app. 1.4 million) to the outer shelf break at 55 km, and were therefore classified into four zones (modified from Moll, 1983; Figure 1). The inshore site is characterized by greatly reduced water clarity and is frequently exposed to discharge from rivers and effluents from the city of Makassar (Moll, 1983; Renema and Troelstra, 2001; Sawall et al., 2011). Near-shore and midshelf sites only receive additional effluent loads during times of increased riverine inputs in monsoon seasons (Cleary et al., 2005). All other sites are only affected while experiencing extreme rain events. All islands with one exception are inhabited. Only the outer shelf site at 27 km is a submerged reef platform. We standardized the sampling by always sampling during high tide in the morning hours and by choosing a site in the northwestern area of each island, the area of highest reef accretion.

### Environmental and water quality parameters

The environmental parameters (temperature, salinity, and pH) and chlorophyll *a* were measured with a Eureka Manta 2 multiprobe (*Measurement Specialities, Hampton, USA*). For the water quality analyses five replicate water samples were taken from 5 m water depth (which was ~1 m above the substrate) with a 5 l Niskin bottle (HydroBios, Kiel, Germany). From each replicate subsamples were taken for measurement of the water quality parameters (inorganic nutrients, DOC, TEP, and SPM). Samples were stored at −20°C in the dark until analysis immediately after returning to the field station. The longest travel time was no more than 3 h. Inorganic nutrients (nitrite, nitrate, phosphate, and silicate) where measured spectro-photometrically with a Flowsys continuous flow analyser (*Unity scientific, Brookfield, USA*). For DOC, samples were filtered through 0.45 μm pore Whatman GF/F filters (*Whatman, GE Healthcare, Pittsburgh, USA*) and acidified with HCl (pH below 2). The measurement was completed via high-temperature oxic combustion (HTOC) using a TOC-VCPH TOC analyzer (*Shimadzu, Mandel, Canada*). Hansell artificial seawater standards (Hansell laboratory RSMAS, University of Miami) and ultrapure water blanks were used for calibration and quality control. To determine SPM mass, pre-combusted GF/F filter were weighed on a ME 36S balance (*Sartorius, Göttingen, Germany*) before and after filtration of known volume of sample water. Difference in weight was determined after filters were dried for 24 h at 40°C. TEP was quantified with an updated protocol (Engel, 2009) of the spectrophotometric method first introduced by Passow and Alldredge (1995). In short this method relates the adsorption of a dye to the weight of polysaccharides filtered on 0.4 μm polycarbonate filters. To relate the absorbance measured on the filters to a reference polysaccharide we prepared a calibration curve by filtering and staining different volumes of Gum Xanthan from *Xanthomonas campestris* cultures.

### Bacterial community analyses

From each site, a 1 L subsample was filtered sequentially using 3 and 0.2 μm Whatman Nuclepore polycarbonate filters (*Whatman, GE Healthcare, Pittsburgh, USA*), to separate bacterioplankton in two selected size fractions representing “particle-attached” and “free-living” bacteria, respectively. DNA extraction for water column samples followed the protocol established by Boström et al. (2004) without modification. In short, cells collected onto filters were lysed in two steps of lysozyme and proteinase K treatment. DNA was recovered using sodium acetate and isopropanol precipitation. Surface sediment was collected from the uppermost 1 cm of sediment at each site. Sediment samples were allocated to 2.0 ml tubes (Eppendorf, Germany) and stored at −20°C until extraction. Coral mucus samples were obtained from individual corals of the genus *Fungia* collected at the same depth by exposing them to air for about 1 min to stimulate the mucus secretion. DNA extraction for sediments and mucus were conducted using the PowerSoil™ DNA Isolation Kit (MO BIO Laboratories, Carlsbad, USA) with modification of two steps of the protocol: (1) we did not incubate for 5 min at 4°C but went straight for the centrifugation and (2) we used 50 μl of elution buffer instead of 100 μl. Extracted DNA samples were sequenced, after 16S rRNA amplification (PCR), Roche/454-tagging and preparation of the Pico Titer Plate, on a Genome Sequencer FLX System + Titanium (Roche, Basel, Switzerland) by LGC Genomics (Berlin, Germany). 16S rRNA primers 341F (5′-CCTACGGGNGGCWGCAG-3′) and 1061R (5′-CRRCACGAGCTGACGAC-3′) were used, targeting the V3–V6 hypervariable region (Ong et al., 2013). Sequences were analyzed in mothur (Schloss et al., 2009), following the standard operating procedure for 454 data with the following exceptions: After pyronoise removal in mothur, primer sequences were removed using cutadapt (Martin, 2011). Only sequences, which contained the forward primer, were considered for the further analysis. After the alignment of the sequences against the SILVA seed reference database (release 123) provided by mothur, we did not perform a preclustering step as our data set was small enough to proceed with the analysis without high computational costs. Operational taxonomic units (OTUs) were defined at a sequence similarity cut-off of 97% using average linkage hierarchical clustering. Unclassified sequences as well as sequences affiliated with mitochondria, chloroplasts, archaea, and eukaryotes were removed from the data set. An overview of the bioinformatic sequence analysis is provided in Supplementary Table 2. The taxonomic classification of representative sequences of OTUs affiliated with potentially pathogenic bacterial taxa was further curated using NCBI blastn against the 16S ribosomal database (date accessed: 19.10.2016). Sequence data is available at the European Nucleotide Archive (ENA), accession no. PRJEB18641.

For cell enumeration staining with 4′6-diamidino-2-phenylindole (DAPI) for epifluorescence microscopy was conducted by incubating filter slices with 20 μl of a 1 μg ml^−1^ DAPI solution for 5 min in the dark. Afterwards they were washed with 80% ethanol, rinsed with distilled water and subsequently dried for 30 min. in the dark. Then 30 μl 4:1 Vecta shield and glycerin solution was added (Vector Laboratories, Burlingame, USA) before enumeration. Ten fields of view were counted at 1,000x magnification from triplicate slices for each sample.

### Statistical analysis

Differences among sites in environmental parameters and water quality were analyzed with SigmaPlot 13.0 software (*Systat Software, Inc., San Jose, California, USA*). Values are given as arithmetic mean ± standard deviation. All parameters failed the Shapiro-Wilk test for normal distribution, so alternative non-parametric Kruskal-Wallis ANOVA on ranks was performed. Whenever significant differences were detected, pair-wise comparisons were conducted using the implementation of the Tukey's HSD *post-hoc* test for non-parametric data in SigmaPlot. Principle component analysis (PCA), including all environmental and water quality parameters, was conducted using the PRIMER 6.16 software (Clarke and Gorley, 2006). Additionally, the effect of sampling site, i.e., the distance to Makassar on each parameter was tested separately with Kruskal Wallis tests and spearman correlations.

Alpha diversity of the bacterial communities was assessed using Hill numbers (Chao et al., 2014). The Hill numbers *q* = 0 (number of OTUs), *q* = 1 (exponential Shannon index), and *q* = 2 (inverse Simpson index) were calculated based on 100 repeated subsampling runs to rarefy the data set to the minimum library size (191 sequences). Kruskal-Wallis and spearman correlations were conducted to test the effect of reef habitat and distance to Makassar on bacterial alpha diversity, respectively. Differences in bacterial community composition (beta diversity) among reef habitats and with increasing distance to Makassar were tested using PERMANOVA in combination with ANOSIM.

If not stated otherwise, statistical analyses were conducted in R using the R core distribution (R Core Team, 2015) and the R package vegan (Oksanen et al., 2016).

## Results

### Spatial variation of environmental and water quality parameters

**Figure 2:**
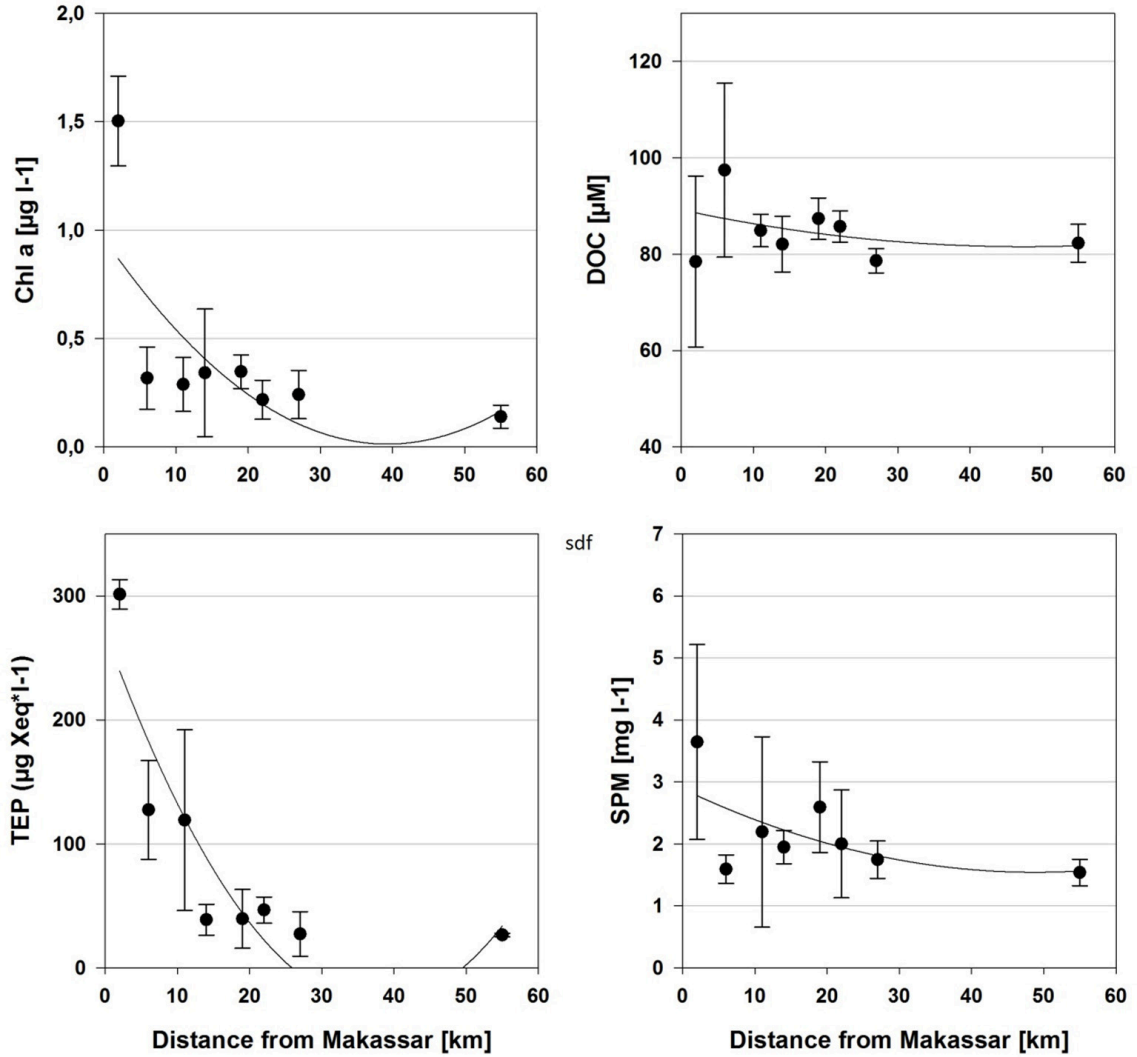
**Mean (±***SD***) concentrations of the selected water quality parameters that were sampled along the hypothesized cross-shelf gradient from the island closest to Makassar (2 km) to the most distant island on the outer shelf (55 km)**. Best-fitting regressions were included. Correlation to distance was strongest for TEP (*r*^2^ = 0.75), followed by chlorophyll a (*r*^2^ = 0.41), SPM (*r*^2^ = 0.13), and DOC (*r*^2^ = 0.04).

The individual islands differed significantly in many of the sampled environmental and water quality parameters, including chlorophyll *a*, TEP, silicate and combined nitrite and nitrate (NO_x_), as revealed by Kruskal-Wallis ANOVA (Table 2). However, Spearman correlations were often low, indicating only a weak monotonous relationship with the distance from Makassar. Furthermore, based on correlations there was no observable trend in environmental parameters (Salinity, temperature, pH) or inorganic nutrients (PO_4_, NO_x_) across the surveyed gradient from the inshore site to the outer shelf break (Tables 1, 2). Only for silicate there was additionally a strong negative correlation (Spearman's ρ −0.83) with increasing distance from Makassar. In contrast to inorganic nutrients, all measured organic nutrient parameters showed changes associated with distance from shore (Figure 2). Chlorophyll *a* exhibited significantly higher concentrations inshore (1.50 ± 0.21 μg l^−1^) compared to the furthest outer shelf site (0.14 ± 0.05 μg l^−1^), but overall correlation to distance from Makassar remained weak (Spearman's ρ −0.44). No significant differences were found in SPM concentrations although there was a weak general decreasing trend from inshore to the outher shelf (Spearman's ρ −0.28). There were significant differences between the highest concentrations of DOC (97.44 ± 17.76 μmol l^−1^) at the closest near-shore station and lowest concentrations, 78.46 ± 18.04 μmol l^−1^, at the inshore station. DOC concentrations between these two zones were also significantly different. Although cross-shelf concentrations of DOC were significantly different (*P* < 0.01), there was only a very low correlation to the distance from Makassar (Spearman's ρ −0.22). Overall chlorophyll *a*, TEP and SPM showed a very similar pattern with steeply declining concentrations between the inshore and the first near-shore site. Near-shore concentrations of TEP were significantly (*P* < 0.02) higher (301.38 ± 11.98 μg Xeq l^−1^) than the most distant outer shelf site (26.34 ± 1.43 μg Xeq l^−1^), and the correlation to distance from Makassar was strong (Spearman's ρ −0.83). The two near-shore sites showed intermediate concentrations while all following stations were in a comparable range to the outermost site.

**Table 1:** **Mean values for the measured environmental and water quality parameters ± ***SD*** across the shelf gradient**.

	**Inshore**	**Near-shore**		**Mid-shelf**			**Outer shelf**	
	**Lae Lae (1 km)**	**Samalona (6 km)**	**Barrang Lompo (11 km)**	**Bonetambung (14 km)**	**Badi (19 km)**	**Lumu Lumu (22 km)**	**K. Kassi (27 km)**	**Kapoposang (55 km)**
Temp. (°C)	29.81 ± 0.03	29.92 ± 0.05	30.04 ± 0.02	30.08 ± 0.05	29.7 ± 0.02	30.09 ± 0.03	29.41 ± 0.02	29.84 ± 0.05
Salinity	32.2 ± 0.02	31.8 ± 0.12	32.1 ± 0.03	32.2 ± 0.03	31.9 ± 0.03	32.3 ± 0.02	31.9 ± 0.02	31.6 ± 0.02
pH	8.16 ± 0.01	8.2 ± 0.01	8.17 ± 0.01	8.21 ± 0.01	8.18 ± 0.01	8.20 ± 0.00	8.17 ± 0.00	8.21 ± 0.01
BOD water column (mg l^−1^ h^−1^)	0.008 ± 0.006	0.041 ± 0.008	0.010 ± 0.006	0.016 ± 0.002	0.011 ± 0.002	0.012 ± 0.004	0.020 ± 0.005	0.020 ± 0.003
BOD surface sediment (mg l^−1^ h^−1^)	0.036 ± 0.007	n/a	0.093 ± 0.013	0.016 ± 0.003	0.036 ± 0.011	0.009 ± 0.002	0.022 ± 0.003	0.005 ± 0.015
BOD 3 cm sediment (mg l^−1^ h^−1^)	0.030 ± 0.001	n/a	0.053 ± 0.012	0.018 ± 0.002	0.041 ± 0.010	n/a	0.031 ± 0.003	0.005 ± 0.002
NOx (μM)	0.46 ± 0.13	0.40 ± 0.05	0.48 ± 0.09	0.13 ± 0.03	0.25 ± 0.08	0.16 ± 0.03	0.48 ± 0.05	0.27 ± 0.03
PO4 (μM)	0.11 ± 0.01	0.09 ± 0.01	0.09 ± 0.01	0.08 ± 0.01	0.08 ± 0.01	0.08 ± 0.01	0.11 ± 0.01	0.09 ± 0.01

**Table 2:** **Kruskal Wallis tests and spearman correlations on effect of sampling site, i.e., the distance to Makassar on each water quality parameter**.

	**Rho**	***p***	**Significance**
DOC	−0.22	1.25E-01	ns
NO_x_	−0.41	3.38E-03	^**^
PO_4_	−0.27	5.39E-02	ns
Si	−0.83	5.32E-14	^***^
SPM	−0.28	6.60E-02	ns
TEP	−0.83	6.09E-07	^***^
Chl a	−0.42	2.29E-12	^***^
pH	0.36	4.32E-09	^***^
Salinity	−0.33	4.03E-08	^***^
Temp. (°C)	−0.22	4.76E-04	^***^

ns, non-significant; *p < 0.05;

** p < 0.01;

*** *p < 0.001*.

**Figure 3:**
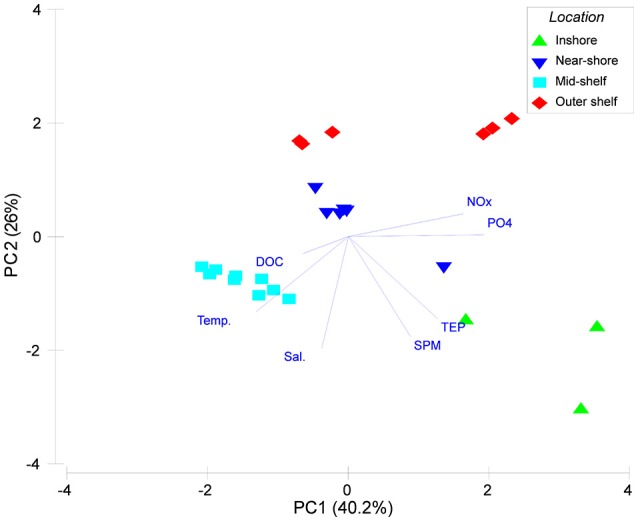
**Principal component (PC) analysis of environmental and water quality parameters for the islands included in the cross-shelf gradient**. Green triangles show samples from the inshore site, blue inverted triangles samples from the inshore area, turquoise squares mid-shelf sites, and red diamonds indicate samples from the outer shelf sampling stations.

A PCA of the environmental and water quality data was able to capture 66.2% of the variation among sites on the first two principal components (Figure 3). The inshore site separates clearly from near-shore and mid-shelf sites by the first principal component. A second group of offshore sites separated on the second PC largely driven by lower salinity and pH-values. This was confirmed by the hierarchical clustering (Supplementary Image 1) where there was low similarity between the inshore island and the remaining sites. Within those remaining islands 2 additional larger clusters formed. Samalona and Barrang Lompo, both categorized as near-shore islands showed a very high similarity (Supplementary Image 1). Additionally, all mid-shelf islands formed a distinct group significantly different from the other groups.

Biological oxygen demand of the water column and sediments showed high variability and did not exhibit a clear pattern across the gradient (Table 1). Within the water column the highest bulk oxygen demand was measured at the 6 km station (0.041 ± 0.008 mg l^−1^ h^−1^) and the lowest at 11 km distance from Makassar (0.010 ± 0.008 mg l^−1^ h^−1^). For both surface and 3 cm deep sediments the highest oxygen uptake was measured at the 11 km site (0.093 ± 0.013 and 0.053 ± 0.012 mg l^−1^ h^−1^, respectively), while lowest BOD was measured at the outer shelf site at 55 km (0.005 ± 0.015 and 0.005 ± 0.002 mg l^−1^ h^−1^, respectively).

### Spatial variation of bacterial communities

No DAPI counts were conducted for the water column of the furthest outer shelf station and the sediments. DAPI cell counts of the combined water column bacterial abundance ranged from 4.39 × 10^6^ ± 4.70 × 10^6^ cells ml^−1^ in the outer shelf area to 7.94 × 10^6^ ± 1.08 × 10^6^ cells ml^−1^ at the inshore site (Supplementary Table 1). At the inshore site compared to midshelf sites 3.23 × 10^6^ ± 2.66 × 10^5^ cells ml^−1^ to 5.87 × 10^6^ ± 5.45 × 10^5^ were counted, respectively. DAPI cell counts for the particle-attached fraction ranged from 1.02 × 10^6^ ± 7.44 × 10^5^ cells ml^−1^ within the midshelf sites to 2.07 × 10^6^ ± 5.22 × 10^5^ cells ml^−1^ at the inshore site. Bacterial abundance in the *Fungia* mucus ranged from 3.34 × 10^7^ ± 2.58 × 10^6^ cells ml^−1^ found within the near-shore area to 9.61 × 10^7^ ± 7.88 × 10^6^ cells ml^−1^ at one of the midshelf sites (Supplementary Table 1).

The molecular analysis of the bacterial communities with 454 Pyrosequencing yielded on average 1051 ± 496 and 1392 ± 110 sequences after quality control per station for the 0.2 μm and 3.0 μm size fractions of the water column, respectively (Supplementary Table 1). For the investigated reef sediments we identified an average of 822 ± 90 sequences at each station, while there was an average of 1,081 ± 558 sequences per station in the *Fungia* mucus. Alpha diversity indices (e.g., Hill *q* = 2, Supplementary Table 1) were lowest in the free-living fraction of the water column (from 2.49 ± 0.02 to 6.18 ± 0.04) and *Fungia* mucus (from 3.65 ± 0.03 to 5.6), followed by the particle-attached fraction of the water column (from 3.68 ± 0.03 to 11.54 ± 0.09) and sediments (from 87.10 ± 1.37 to 156.48 ± 0.85). Bacterial communities in the reef sediments were generally more diverse (Supplementary Table 1) and the taxa were more evenly distributed compared to the coral mucus and water column. Rarefaction curves of the sediment, contrary to water column and mucus samples, still increased steeply (Supplementary Image 2). This indicates that some fraction of the diversity was not covered due to the comparatively low number of sequences obtained from the sediments.

**Figure 4:**
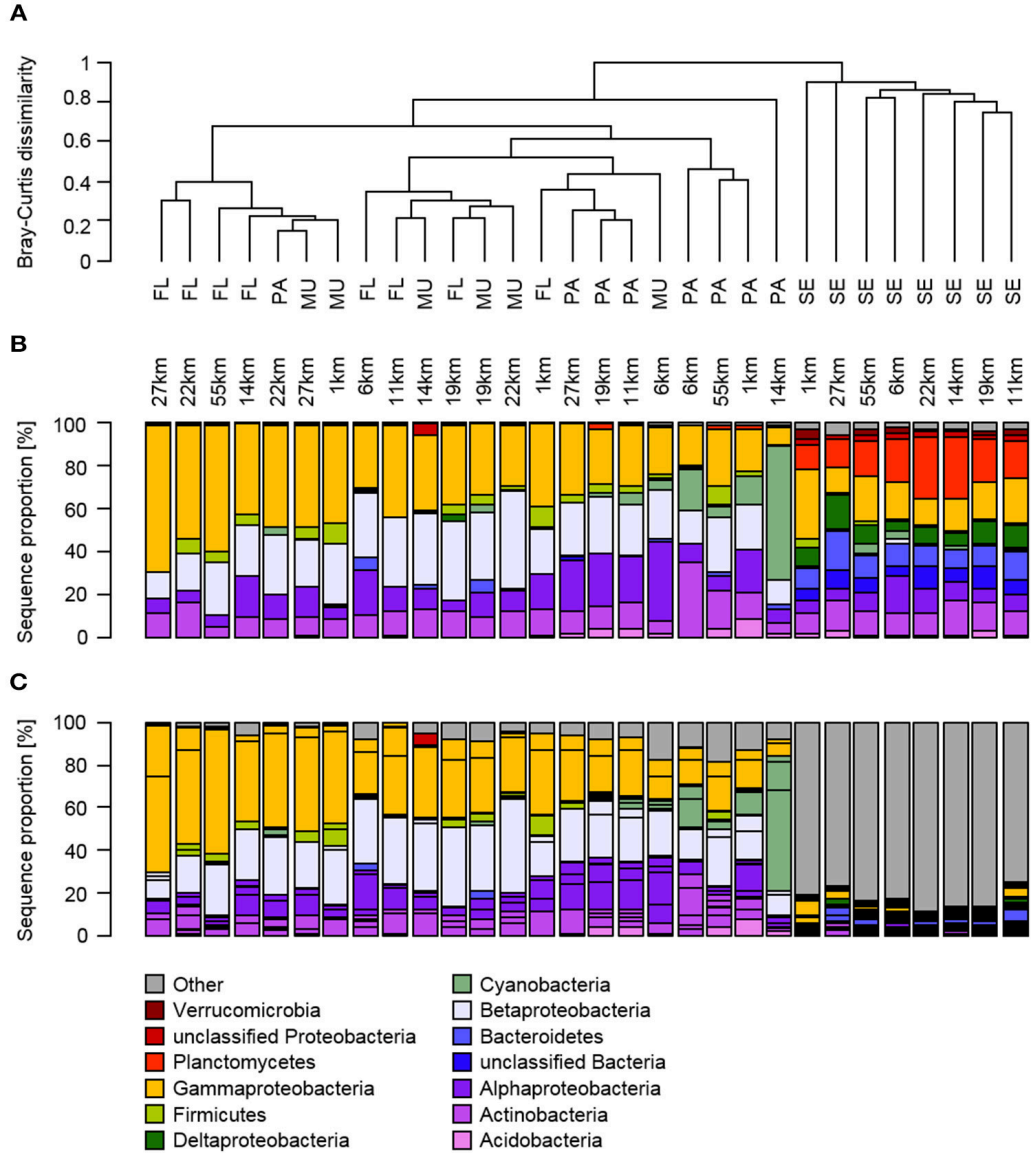
**Relative abundance of bacterial phyla (B: top bar chart) and OTUs (C: bottom bar chart) in the free-living, particle-attached fraction of the water column and from ***Fungia*** mucus and sediments from inshore to outer shelf reef sites, as well as the hierarchical clustering based on Bray-Curtis dissimilarities (A: top part), of the investigated habitats according to their location on the shelf**. The Proteobacteria in **(B)** are further identified to class level to accommodate for the high diversity and occurrence of biogeochemically important classes within that phylum.

The composition of the bacterial communities in the sediment was distinct from the other habitats based on the clustering of Bray-Curtis dissimilarity coefficients (Figure 4A). This was confirmed by a PERMANOVA analysis, which revealed significantly different communities between the habitats [*R*^2^ = 0.47, *F*_(3, 29)_ = 7.90, *P* < 0.001], but not with distance to Makassar [*R*^2^ = 0.03, *F*_(3, 29)_ = 1.36, *P* > 0.05]. Subsequent pairwise tests showed that all habitats, except the *Fungia* mucus compared with both size fractions of the water column, were significantly different from each other (ANOSIM, *P* < 0.05).

### Water column

**Figure 5:**
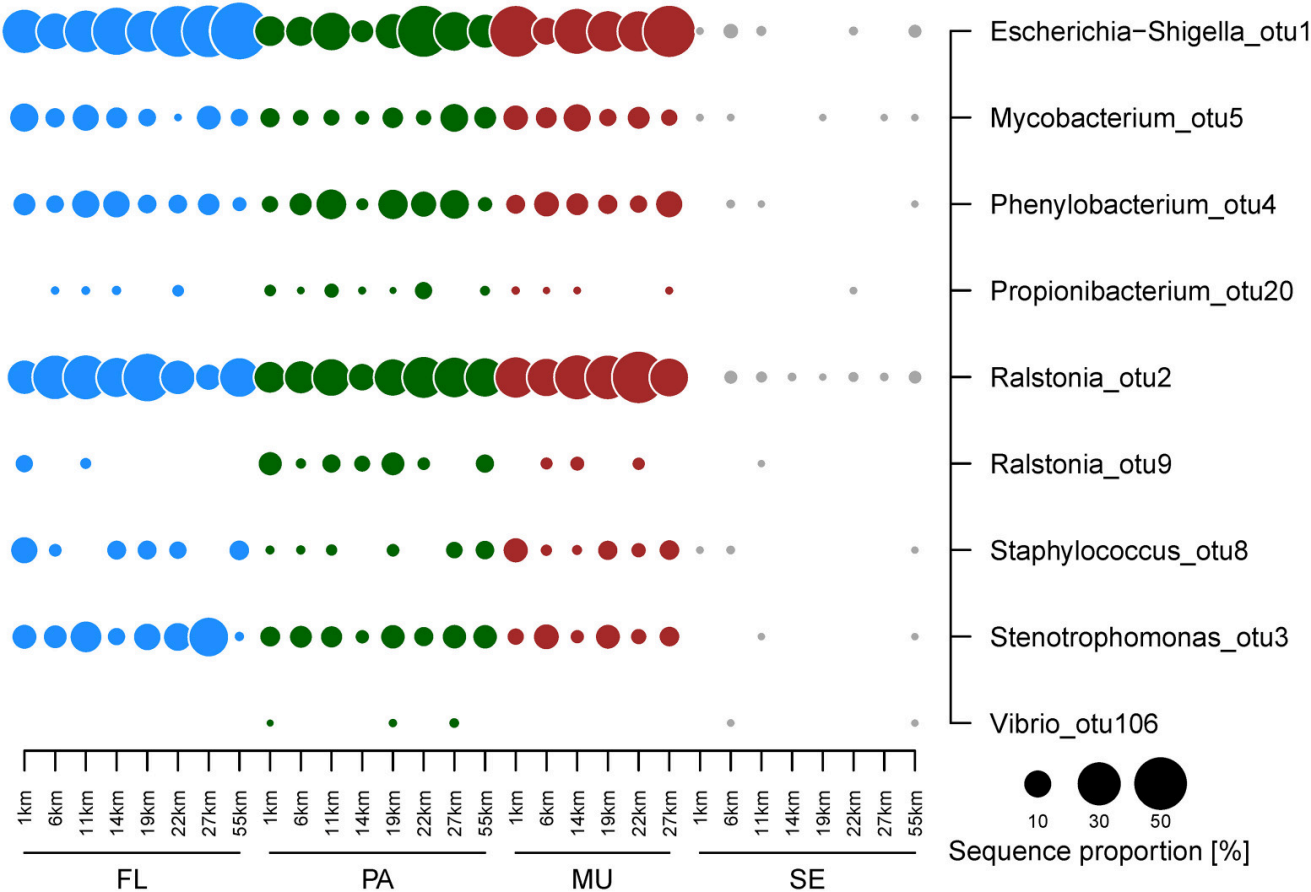
**Relative abundance of potentially pathogenic bacterial OTUs identified in the free-living (green circles), particle-attached (blue circles) fraction of the water column, as well as ***Fungia mucus*** (red circles) and sediments (gray circles)**. OTU numbers indicate decreasing ranked total abundance in the data set.

The free-living fraction of the water column bacterial communities showed variation among stations across the spatial gradient (Figure 4). Three classes constituted the majority of all identified bacteria of the free-living fraction of the water column: the Alpha-, Beta-, and Gammaproteobacteria (Figure 4B). The relative abundance of Gammaproteobacteria ranged from 29% at the 6 km sampling station to 69% at the stations on the outer shelf site at 27 km (Figure 4). In contrast, Alphaproteobacteria showed highest sequence proportions (22%) at the inshore site at 6 km, and decreased toward the mid-shelf sites (lowest relative abundance 5% at 19 km). Betaproteobacteria also showed no obvious trends in changing relative abundances across the gradient, and their relative contribution to the total community varied from 13–38%. Within those three main classes four OTUs, all of them potential human pathogens, displayed a large contribution to the community composition of the free-living fraction of the water column: *Escherichia*/*Shigella* (Gammaproteobacteria), *Ralstonia* (Betaproteobacteria), *Stenotrophomonas* (Gammaproteobacteria), and *Phenylobacterium* (Alphaproteobacteria; Figure 5). The relative abundance of *Escherichia*/*Shigella*, the causative agent for shigellosis, ranged from 20% at the 6 km sampling station to 58% at the stations on the outer shelf site at 55 km (Figure 5). *Ralstonia* showed highest concentrations (37%) at the mid-shelf site at 11 km, and decreased toward the outer shelf sites (8% at 19 km). *Stenotrophomonas* and *Phenylobacterium* showed no obvious trends across the gradient, and their relative contribution to the total community varied from <1 to 24 and 2 to 10%, respectively. Other potentially pathogenic bacterial groups that were found among the 10 most dominant OTUs were *Mycobacterium*, the causative agent of tuberculosis and leprosy, and *Staphylococcus* (Firmicutes). There was no overall trend in the abundance of the potentially pathogenic OTUs with distance from Makassar. While e.g., *Escherichia*/*Shigella* increased in abundance with distance, *Mycobacterium* (Actinobacteria) is most abundant at the very inshore site closest to Makassar.

*Escherichia*/*Shigella* and *Ralstonia* were, on average, also the dominant OTUs that were retained on 3.0 μm filters, and thus being defined as “particle-attached.” The highest relative abundance of *Escherichia*/*Shigella* was found at the 22 km site (44%), while the lowest abundance was observed at the 14 km site (6%). *Ralstonia* was generally found in lowest abundance at 14 km (10%, Figure 4). The relative abundance was highest at the mid-shelf site at 22 km (27%). The cyanobacterium *Synechococcus* was the third most abundant OTU observed in the particle-attached fraction, with an average abundance of 10%. This OTU was not detected in any but one sample of the free-living water fraction at a relative abundance <1%. The prevalence of potentially pathogenic OTU, including *Escherichia*/*Shigella, Ralstonia, Phenylobacterium*, and *Mycobacterium*, were again high. For potentially pathogenic, particle-attached bacteria, there was no overall pattern in relative abundance across the gradient. There were significant but weak differences in bacterial community composition between the “free-living” and “particle-attached” size fraction (ANOSIM P 0.003, R 0.25). Calculated Bray-Curtis dissimilarities were also often >50% (Figure 4A).

### Fungia mucus

Overall, the bacterial community composition of the mucus samples showed no statistically significant differences to both water column size fractions. On the class level Alpha-, Beta-, and Gammaproteobacteria were again the most dominant taxa. The Gammaproteobacteria contributed to 22–49% of the total bacterial community, Betaproteobacteria 22–46% and Alphaproteobacteria 6–37% (Figure 4B). There was no pattern in the changes of the relative abundance across the gradient. *Escherichia*/*Shigella* and *Ralstonia* were also the two dominant OTU in the *Fungia* mucus (Figure 4C). They alternated as the single most dominant OTU along the gradient. *Escherichia*/*Shigella* contributed to 10–44% of the total bacterial community, *Ralstonia* 6–37% and *Phenylobacterium*, as the third most abundant OTU, ranged from 3–9%. Members of the also potentially pathogenic OTU *Staphylococcus, Propionibacterium*, and *Mycobacterium* were detected at similar relative abundances throughout the observed gradient (Figure 5).

### Reef sediments

Overall the sediment communities were more diverse and even in their community composition and showed less variation across the shelf on the class level (Supplementary Table 1, Figure 4C). Some classes were almost exclusively found in the sediment, including Planctomycetes (11–29%), Bacteroidetes (8–18%), and Deltaproteobacteria (5–16%, Figure 4). From the identified classes only Gammaproteobacteria exhibited stronger changes in relative abundance across the gradient, with higher relative OTU abundance (32%) at the inshore site at 1 km, which is continuously affected by effluents from urban Makassar, compared to further off-shore (lowest relative abundance observed at 27 km, 11%). In contrast to the mucus and water column samples, there was also no dominance of few individual OTUs in the bacterial community. The OTUs with highest average contribution to total relative abundance were *Muriicola* (2%, Bacteroidetes), and unclassified members of the gammaproteobacterial JTB255 marine benthic group (2%) and the firmicute family Carnobacteriaceae (1%). Additionally, the relative contribution of the investigated potentially pathogenic OTUs to the overall community composition was much lower (Figure 5).

## Discussion

With this study we can shed new light on shifts in bacterial communities of the water column, reef sediments and coral mucus in response to coastal eutrophication. Similar to other previous investigations we found strong changes in water quality parameters along the gradient (Sawall et al., 2011). But effects could only be detected for the inshore site and were limited to a few parameters, namely Si, chlorophyll *a*, and TEP. Although SPM showed a very similar pattern, the differences were not significant. While there were no significant effects of the water quality parameters and distance from Makassar on the bacterial community composition we detected distinct and significant differences between the habitats. Overall, the sediment bacterial communities were more diverse and heterogeneous, comparable to previous descriptions of carbonate-based reef sediments (Hewson et al., 2003). Moreover, while Gammaproteobacteria was the most dominant class in the water column and *Fungia* mucus, Planctomycetes was the most abundant class in the reef sediments.

### Environmental parameters

Excluding the highly eutrophied inshore site, concentrations of chlorophyll *a*, TEP and SPM in the Spermonde Archipelago where within the range of other near-shore reef ecosystems of the western Pacific, e.g., the Great Barrier Reef, Australia (Alongi et al., 2015), New Caledonia (Fichez et al., 2010) or, for chlorophyll *a*, previous studies of the same area (Sawall et al., 2011). Interestingly, the effect of riverine and urban sewage is limited only to the inshore site. One possible reason is a dilution effect by a strong longshore current in the Spermonde Archipelago. A large proportion of the Indonesian through-flow, connecting the Pacific with the Indian Ocean, is channeled through the Makassar Strait, leading to a constant southward flow of water (Gordon et al., 2003). As concentrations of chlorophyll *a* and the severity of eutrophication-related processes were highly correlated to water residence time (Delesalle and Sournia, 1992; Howarth et al., 2011), the rapid flushing can have a dilution effect on the measured parameters. This is also the case in the rainy season, when increased riverine input delivers more nutrients to the inshore and near-shore sites. However, since the predominant current through the Makassar Strait is southward, the river plume of the Jene Berang river is also deflected south and only reaches out to the closest near-shore station (Renema and Troelstra, 2001). In both cases, excess nutrients will not be available for phytoplankton growth and bacterial production in areas north of the deflected plume, e.g., the mid-shelf and outer shelf sites.

TEP concentrations can be used as a good integrative indicator for differences in the water quality among the sites as well as for a strong interaction between phytoplankton and the bacterial communities, which results in TEP production (Smith et al., 1995; Gärdes et al., 2011). TEP was strongly correlated to chlorophyll *a*, SPM concentrations and DAPI cell counts in the water column. Additionally, TEP may also serve as a food source and habitat for bacteria (Passow, 2002), increase particle aggregation and subsequent sedimentation (Gärdes et al., 2011; Cárdenas et al., 2015).

Concentrations of DOC in the Spermonde Archipelago are at the higher end described for tropical reef ecosystems (Dinsdale et al., 2008; Nelson et al., 2011), but they do not follow the general decreasing trend, with increasing distance to Makassar, observed for chlorophyll *a*, TEP, and SPM. This could be explained through a combination of DOC rapidly being take up by heterotrophic bacteria in the water column and aggregation into larger particles (Passow and Alldredge, 1994; Passow, 2000; Nelson et al., 2011).

### Spatial variation of bacterial communities

On the community level there were no corresponding shifts along the measured water quality gradients. In the present study, e.g., significantly higher bacterial cell counts in the water column were observed at the inshore site at 1 km distance to Makassar. This can be the result of an increased uptake of available DOC at the inshore site (Ferrier-Pagès et al., 1998). With an increased availability of nutrients and organic matter, the expected shift would be toward a community dominated by heterotrophic bacteria specialized in assimilating the available organic matter. Interestingly, this pattern was not reflected in the sequencing data of neither the free-living nor the particle-attached fractions of the water column. Likely there is a time lag between the additional availability of food sources and the response in the bacterial community. Together with the low water residence time in the Spermonde Archipelago, driven by strong southward water flow due to the Indonesian through-flow (Gordon et al., 2008), this can lead to a shifted pattern in community composition. In support of that, there was a much higher relative abundance of Gammaproteobacteria at the outer shelf sites compared to the eutrophied inshore station. Within that class, *Escherichia/Shigella* was the dominant OTU. Bacteria of this genus are known human pathogens, as well as indicators for drinking water quality and fecal contamination (Baudišová, 1997; Edberg et al., 2000; Odonkor and Ampofo, 2013), and their increased abundance can be attributed to human impact, likely from urban Makassar. Some of the other potential pathogenic taxa, e.g., *Mycobacterium* and *Staphylococcus*, were found at highest relative abundances closer to the mainland. This is noteworthy, as they thrive well in eutrophic conditions (Jacobs et al., 2009), or in areas with high human population density (Yoshpe-Purer and Golderman, 1987). All of the abovementioned taxa also contain OTUs that are capable of degrading petroleum, another source of human coastal pollution (Roy et al., 2002). Among the Alphaproteobacteria, which were most abundant in the water column of the chlorophyll-rich inshore waters of the Spermonde Archipelago, are taxa known to correlate well to waters rich in inorganic nutrients and phytoplankton biomass (Allers et al., 2007; Teira et al., 2008). Although some of the most common oligotrophic bacteria, such as members of the *SAR11/Pelagibacter* clade (Tout et al., 2014; West et al., 2016), belong to the Alphaproteobacteria class, those taxa were only rarely found in the present investigation. There was no observable gradual change in Betaproteobacteria occurrence across the shelf in the Spermonde Archipelago. Betaproteobacteria in general, and OTUs from the genus *Ralstonia* as the main constituents of that class, are very common to freshwater (Lau et al., 2013). There are likely overriding local effects at the individual study sites, such as freshwater seepage from the densely populated islands, which are additionally lacking waste water treatment facilities (Ferse et al., 2012; Williams et al., 2014). This indicates a local source of organic matter for Betaproteobacteria from island effluents. This is further corroborated at the only uninhabited site, a submerged reef platform in the mid-shelf area, which showed the lowest relative abundance in Betaproteobacteria/*Ralstonia* in free-living and particle-attached habitats.

Observations made on the community composition of the mucus samples were similar to those of the water column, which was reflected in the non-significant differences in overall community composition. Again, Gamma- and Betaproteobacteria were on average the most dominant classes. *Escherichia/Shigella* was again the single most common OTU in the mucus samples. This is an unusual observation, as members of the *Escherichia/Shigella* taxon are usually not present in mucus samples in such high relative abundances (Koren and Rosenberg, 2006). This may indicate that the *Fungia* corals at the sampled islands and their fragile host-symbiont balance are already severely disturbed potentially enabling *Escherichia/Shigella* to colonize the coral mucus and capitalize on the available carbon sources. Findings concerning the Betaproteobacteria from the present study are supported by comparisons of bacterial communities in mucus of *Fungia* sp. from the Red Sea and aquaria by Kooperman et al. (2007). They also found increased occurrences of Betaproteobacteria in the aquaria samples with altered water quality. They are also known to correlate strongly to the availability of organic matter in the marine environment (Tada et al., 2011).

The highly diverse reef sediment community was, on the class level, dominated by Planctomycetes, Bacteroidetes, and Gammaproteobacteria, particularly at the inshore site closest to Makassar. Planctomycetes are common colonizers of marine sediments, and are commonly found in anaerobic, high-nutrient conditions and are effective metabolizers of DOC (Chipman et al., 2010; Lage et al., 2012). For the Spermonde Archipelago this implies a high load of organic matter which is deposited to the sediments, as we observed for the nearshore areas during our sampling campaign. Some members of the Bacteroidetes and Gammaproteobacteria also have a large array of extracellular hydrolytic enzymes making them an ecologically and biogeochemically important group in the rapid remineralization of organic matter (Azam and Malfatti, 2007; Dang et al., 2009; Edwards et al., 2010). The highest concentrations of chlorophyll *a*, TEP, and SPM at near-shore sites, indicating a high productivity and anthropogenic impact at the site closest to shore, may support the high prevalence especially of Gammaproteobacteria at this site. As a result from high chlorophyll *a* and SPM concentrations, a constant supply of particles rich in organic matter to the sediments may favor fast-growing, heterotrophic bacteria.

### Inter-habitat variability

Our results suggest that much of the observed variability in bacterial community composition in the Spermonde Archipelago is a result of differences between the sampled habitats. All three habitats, except between the water column and the *Fungia* mucus, showed statistically significant differences in bacterial community composition. Although the two size fractions of the water column were quite similar in the general composition of the most dominant OTU, there were still significant differences in the overall community composition. This is a common observation, also in tropical waters, and often related to differences in food and habitat availability (Zhang et al., 2007; Kellogg and Deming, 2009; Crespo et al., 2013). In case of the Spermonde Archipelago, e.g., *Synechococcus* is almost exclusively found in the particle-attached fraction of the water column.

There is also a frequent exchange between bacterial communities of the water column and the coral mucus, usually controlled by bacteria inhabiting the mucus (Ritchie, 2006). Cell counts in the coral mucus are often 100–1,000 fold higher in the mucus compared to the surrounding seawater, confirming our DAPI cell counts (Rosenberg et al., 2007). Previous studies showed that microbiota associated to a host are often species specific and to a large extent stable across environmental gradients. Barott et al. (2011) showed clear difference in bacterial community composition of different types of algae and corals. Another recent study from the Spermonde Archipelago confirmed this for a variety of reef sponges (Cleary et al., 2015). The production of specific carbohydrates and antibiotics very likely plays an important role in shaping those stable host-specific microbial communities (Ritchie, 2006; Rosenberg et al., 2007). Therefore, bacterial communities from the water column are usually distinct from those inhabiting the mucus (Ritchie and Smith, 2004; Rosenberg et al., 2007), but when that ability of the mucus bacteria fails to select for a host-specific community, e.g., due to anthropogenic stress such as eutrophication or ocean acidification, other opportunistic bacteria might invade available space in the mucus. Therefore, similar bacterial community composition between coral mucus and water-column, as observed as an opportunistic colonization by *Escherichia/Shigella, Ralstonia*, and *Mycobacterium* in the present study, can indicate a failure of control mechanisms for mucus colonization due to stress.

Reef sediments are a biogeochemically very different environment compared to habitats exposed to water column. As observed for the present study, they are also often more diverse than communities from the water column, and the full diversity is rarely assessed (Uthicke and McGuire, 2007; Gaidos et al., 2011). Bacteria inhabiting the sediments provide important functions in the coupling of pelagic and benthic organic matter degradation processes (Gaidos et al., 2011; Schöttner et al., 2011). Due to the high remineralization activity occurring in the sediment-water interface there is usually drop in O_2_ and subsequently available electron acceptors after the first few mm (Rasheed et al., 2004). This leads to functionally very different and diverse communities in the different sediment strata (Gaidos et al., 2011), similar to observations made in the Spermonde Archipelago.

## Conclusions

This study contributes the first conclusive overview of changes in bacterial communities from the water column, sediments, and coral mucus in relation to changes in the water quality of the Spermonde Archipelago. There were significant differences between the investigated habitats, namely between the sediments, coral mucus, and the water column as well as between the two size fractions of the water column. The potentially pathogenic bacterial OTUs *Escherichia/Shigella, Ralstonia*, and *Mycobacterium* were among the most abundant taxa observed in the water column and coral mucus in this study, while there was no dominant OTU in the sediments. Unfortunately, our identification of potentially pathogenic OTUs was only based on high sequence similarity to known pathogenic strains, and we could not test for actual pathogenicity of these OTUs. Further, tests for the functional traits are required to confirm our assumption. Alarmingly, the prevalence of many of the observed, potentially pathogenic bacteria was much higher at the chronically impacted sampling sites closer to urban Makassar. If coastal development and waste water management remains unchanged this may have unpredictable consequences for the coastal and island populations that strongly depend on the natural resources taken from the Archipelago.

## Author contributions

HK, AG, MT, CW, and ML developed the idea for this project. HK conducted the fieldwork with support from JP and AG and logistical support from ML. The statistical analysis was conducted by HK, CH, and JP. HK wrote the manuscript with input and critical revision from all co-authors.

## Funding

This project was part of a Junior Research Group Leader position granted to AG by the Leibniz Association.

### Conflict of interest statement

The authors declare that the research was conducted in the absence of any commercial or financial relationships that could be construed as a potential conflict of interest.

## Supplementary material

Supplementary Image 1 **Hierarchical clustering, based on Bray-Curtis dissimilarities, of water quality parameters in the different shelf areas**.

Supplementary Image 2 **Rarefaction curves for alpha diversity indices (Hill numbers) including obersed sequencing depth and extrapolated for twice the sequencing depth per sample using the iNEXT R package**. Number of OTUs (*q* = 1), exponential Shannon diversity index (*q* = 1), inverse Simpson index (*q* = 2). FL, free-living fraction of the water column; PA, particle-attached fraction of the water column; MU, Fungia coral mucus; SE, sediment.

Supplementary Table 1 **Number of 16S sequences generated on the 454 platform per sample (raw), as well as number of sequences after quality filtering and primer removal (QC), and after alignment, chimera removal and taxonomic classification (Classified)**. OTU numbers are given for the complete data set (nOTU), alpha diversity indices (Hill numbers *q* = 0, *q* = 1, *q* = 2) are provided for the data set rarified to the same library size (191 sequences). Each sample was rarefied 100 times and Hill numbers represent mean ± standard error. FL, free-living fraction of the water column; PA, particle-attached fraction of the water column; MU, Fungia coral mucus; SE, sediment. DAPI cell counts are given in cells ml^−1^ ± standard deviation.

Supplementary Table 2 **Bioinformatic processing of 454 sequences**.
