# Supplementary figures and images for: Bacterial Community Composition and Potential Driving Factors in Different Reef Habitats of the Spermonde Archipelago, Indonesia
Source: Front Microbiol. 2017 Apr 20;8:662. doi: 10.3389/fmicb.2017.00662 (PMC5397486; doi:10.3389/fmicb.2017.00662)

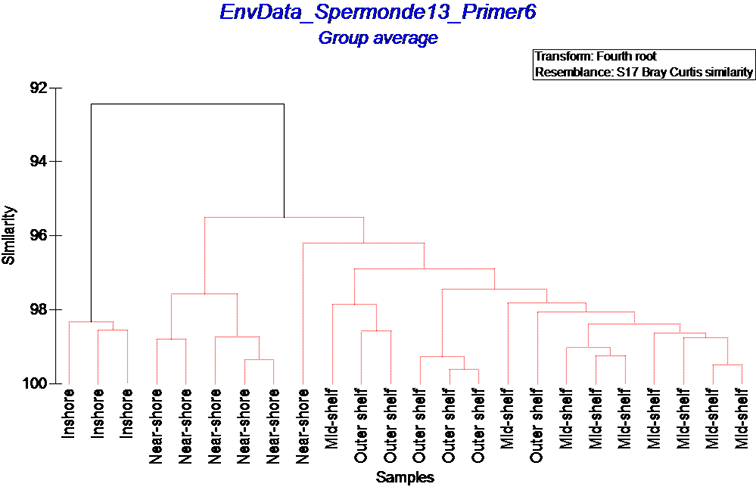

Supplement: Supplementary Image 1 — Hierarchical clustering, based on Bray-Curtis dissimilarities, of water quality parameters in the different shelf areas. [file Image1.TIF]

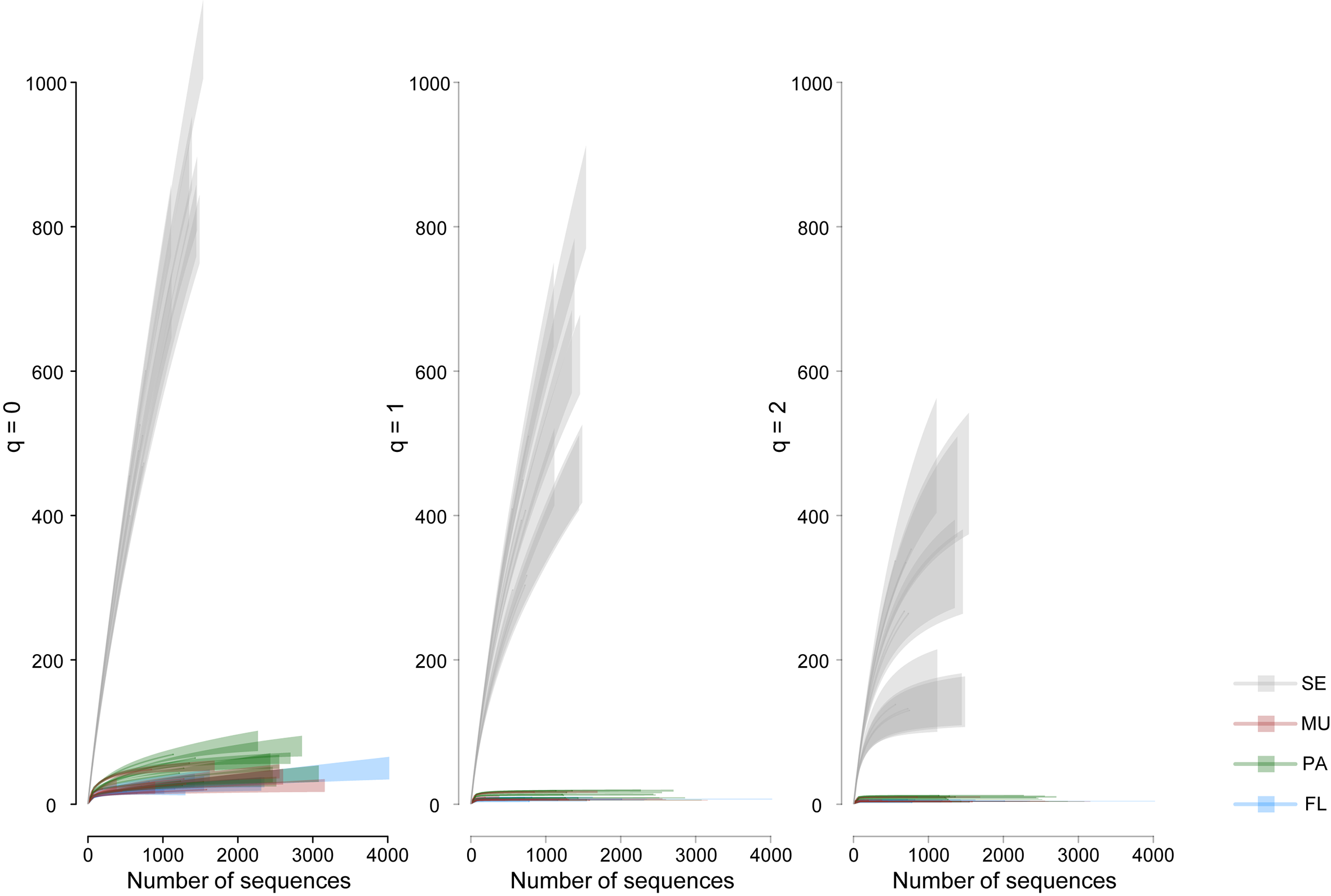

Supplement: Supplementary Image 2 — Rarefaction curves for alpha diversity indices (Hill numbers) including obersed sequencing depth and extrapolated for twice the sequencing depth per sample using the iNEXT R package. Number of OTUs (q = 1), exponential Shannon diversity index (q = 1), inverse Simpson index (q = 2). FL, free-living fraction of the water column; PA, particle-attached fraction of the water column; MU, Fungia coral mucus; SE, sediment. [file Image2.tif]
