# Supplementary material for: Bacterial Community Composition and Potential Driving Factors in Different Reef Habitats of the Spermonde Archipelago, Indonesia
Source: Front Microbiol. 2017 Apr 20;8:662. doi: 10.3389/fmicb.2017.00662 (PMC5397486; doi:10.3389/fmicb.2017.00662)
Supplement: Supplementary Table 1 — Number of 16S sequences generated on the 454 platform per sample (raw), as well as number of sequences after quality filtering and primer removal (QC), and after alignment, chimera removal and taxonomic classification (Classified). OTU numbers are given for the complete data set (nOTU), alpha diversity indices (Hill numbers q = 0, q = 1, q = 2) are provided for the data set rarified to the same library size (191 sequences). Each sample was rarefied 100 times and Hill numbers represent mean ± standard error. FL, free-living fraction of the water column; PA, particle-attached fraction of the water column; MU, Fungia coral mucus; SE, sediment. DAPI cell counts are given in cells ml−1 ± standard deviation. [file Table1.docx]

Supplementary Table 1: Number of 16S sequences generated on the 454 platform per sample (raw), as well as number of sequences after quality filtering and primer removal (QC), and after alignment, chimera removal and taxonomic classification (Classified). OTU numbers are given for the complete data set (nOTU), alpha diversity indices (Hill numbers q = 0, q = 1, q = 2) are provided for the data set rarified to the same library size (191 sequences). Each sample was rarefied 100 times and Hill numbers represent mean ± standard error. FL: free-living fraction of the water column, PA: particle-attached fraction of the water column, MU: Fungia coral mucus, SE: sediment. DAPI cell counts are given in cells ml^-1^ ± standard deviation.

| Site | Habitat | Raw | QC | Classified | nOTU | Hill (q = 0) | Hill1 (q = 1) | Hill2 (q = 2) | DAPI (cells ml^-1^) |
| --- | --- | --- | --- | --- | --- | --- | --- | --- | --- |
| BD (19 km) | FL | 2590 | 1215 | 1177 | 25 | 16 ± 0.15 | 6.61 ± 0.05 | 4.34 ± 0.04 | 3.82E+06 ± 3.28E+05 |
| BD (19 km) | PA | 2798 | 1292 | 1218 | 43 | 24 ± 0.20 | 12.36 ± 0.09 | 8.88 ± 0.07 | 1.02E+06 ± 7.44E+05 |
| BD (19 km) | MU | 465 | 207 | 191 | 21 | 21 | 9.06 | 5.60 | 2.88E+08 ± 2.36E+07 |
| BD (19 km) | SE | 2047 | 922 | 769 | 600 | 173 ± 0.40 | 163.16 ± 0.65 | 146.74 ± 1.21 | NA |
| BL (11 km) | FL | 1749 | 690 | 651 | 15 | 12 ± 0.12 | 5.87 ± 0.03 | 4.64 ± 0.03 | 3.52E+06 ± 3.70E+05 |
| BL (11 km) | PA | 2969 | 1329 | 1233 | 32 | 21 ± 0.18 | 11.51 ± 0.09 | 7.82 ± 0.07 | 1.29E+06 ± 3.43E+05 |
| BL (11 km) | SE | 1919 | 870 | 744 | 472 | 152 ± 0.51 | 126.91 ± 0.86 | 87.10 ± 1.37 | NA |
| BT (14 km) | FL | 1343 | 547 | 510 | 17 | 14 ± 0.11 | 6.87 ± 0.04 | 4.60 ± 0.03 | 3.52E+06 ± 4.37E+05 |
| BT (14 km) | PA | 3651 | 1479 | 1429 | 64 | 25 ± 0.29 | 7.49 ± 0.08 | 3.90 ± 0.04 | 1.18E+06 ± 2.24E+05 |
| BT (14 km) | MU | 3004 | 1433 | 1278 | 35 | 18 ± 0.20 | 6.62 ± 0.06 | 4.37 ± 0.03 | 1.24E+08 ± 1.91E+07 |
| BT (14 km) | SE | 1788 | 846 | 693 | 525 | 171 ± 0.35 | 161.84 ± 0.54 | 147.72 ± 0.94 | NA |
| KK (27 km) | FL | 2189 | 1228 | 1157 | 21 | 12 ± 0.16 | 5.23 ± 0.04 | 3.63 ± 0.03 | NA |
| KK (27 km) | PA | 2864 | 1345 | 1258 | 26 | 16 ± 0.16 | 8.52 ± 0.06 | 6.36 ± 0.05 | NA |
| KK (27 km) | MU | 1065 | 493 | 449 | 18 | 14 ± 0.14 | 5.76 ± 0.04 | 3.83 ± 0.03 | 1.11E+08 ± 6.99E+06 |
| KK (27 km) | SE | 2070 | 898 | 722 | 467 | 148 ± 0.53 | 123.03 ± 0.82 | 88.66 ± 1.12 | NA |
| KP (55 km) | FL | 1780 | 829 | 775 | 21 | 13 ± 0.14 | 3.83 ± 0.03 | 2.49 ± 0.02 | 3.52E+06 ± 4.37E+05 |
| KP (55 km) | PA | 3073 | 1446 | 1352 | 57 | 33 ± 0.23 | 16.45 ± 0.15 | 9.89 ± 0.12 | NA |
| KP (55 km) | SE | 1816 | 869 | 729 | 510 | 164 ± 0.40 | 151.05 ± 0.64 | 131.24 ± 1.08 | NA |
| LL (1 km) | FL | 1874 | 803 | 713 | 25 | 16 ± 0.18 | 8.32 ± 0.06 | 6.18 ± 0.04 | 5.87E+06 ± 5.45E+05 |
| LL (1 km) | PA | 2766 | 1239 | 1135 | 69 | 32 ± 0.28 | 15.99 ± 0.12 | 11.54 ± 0.09 | 2.07E+06 ± 5.22E+05 |
| LL (1 km) | MU | 3875 | 1810 | 1580 | 20 | 12 ± 0.12 | 5.30 ± 0.04 | 3.68 ± 0.03 | 1.44E+08 ± 1.89E+07 |
| LL (1 km) | SE | 1664 | 661 | 558 | 399 | 158 ± 0.44 | 135.72 ± 0.72 | 96.52 ± 1.31 | NA |
| LU (22 km) | FL | 4481 | 2227 | 2010 | 34 | 15 ± 0.17 | 6.50 ± 0.06 | 4.12 ± 0.04 | 3.23E+06 ± 5.66E+05 |
| LU (22 km) | PA | 3117 | 1610 | 1540 | 31 | 15 ± 0.18 | 5.57 ± 0.05 | 3.68 ± 0.03 | 1.17E+06 ± 1.95E+05 |
| LU (22 km) | MU | 2950 | 1396 | 1302 | 28 | 16 ± 0.15 | 5.88 ± 0.05 | 3.65 ± 0.03 | 1.71E+08 ± 1.38E+07 |
| LU (22 km) | SE | 1574 | 686 | 554 | 454 | 175 ± 0.34 | 167.58 ± 0.51 | 156.48 ± 0.85 | NA |
| SL (6 km) | FL | 1905 | 868 | 813 | 31 | 21 ± 0.19 | 9.01 ± 0.07 | 5.91 ± 0.04 | 3.94E+06 ± 3.54E+05 |
| SL (6 km) | PA | 2757 | 1395 | 1276 | 50 | 27 ± 0.23 | 14.52 ± 0.11 | 10.87 ± 0.08 | 1.16E+06 ± 2.00E+05 |
