# Supplementary material for: Bacterial Community Composition and Potential Driving Factors in Different Reef Habitats of the Spermonde Archipelago, Indonesia
Source: Front Microbiol. 2017 Apr 20;8:662. doi: 10.3389/fmicb.2017.00662 (PMC5397486; doi:10.3389/fmicb.2017.00662)
Supplement: Supplementary Table 2 — Bioinformatic processing of 454 sequences. [file Table2.docx]

Supplementary Table 2: Bioinformatic processing of 454 sequences.

| Analysis step | Command | Parameter settings |
| --- | --- | --- |
| Trimming of flow grams | trim.flows | minflows=360  maxflows=720  order=B |
| Denoising  (pyronoise removal) | shhh.flows | Lookup=LookUp_Titanium.pat mindelta=0.001  order=B |
| Primer removal | cutadapt | -O 15  -g CCTACGGGNGGCWGCAG  -e 0.16 |
| Alignment | align.seqs | reference=silva.seed_v123.align  flip=T |
| Trimming of alignment | screen.seqs | optimize=start-end  criteria=95 |
| Chimera detection | chimera.uchime | default |
| Taxonomic classification | classify.seqs | template=silva.seed_v123.align  taxonomy=silva.seed_v123.tax  cutoff=70 |
| Removal of unwanted lineages | remove.lineage | taxon=Mitochondria-Chloroplast-Archaea-Eukaryota-unknown |
| OTU clustering | dist.seqs | cutoff=0.10 |
|  | cluster | default |
|  | make.shared | label=0.03 |
| Consensus taxonomy | classify.otu | label=0.03, cutoff=70, probs=f |
